# Von Willebrand factor in diagnostics and treatment of cardiovascular disease: Recent advances and prospects

**DOI:** 10.3389/fcvm.2022.1038030

**Published:** 2022-12-02

**Authors:** Sergey Kozlov, Sergey Okhota, Yuliya Avtaeva, Ivan Melnikov, Evgeny Matroze, Zufar Gabbasov

**Affiliations:** ^1^Department of Problems of Atherosclerosis, National Medical Research Centre of Cardiology Named After Academician E.I. Chazov of the Ministry of Health of the Russian Federation, Moscow, Russia; ^2^Laboratory of Cell Hemostasis, National Medical Research Centre of Cardiology Named After Academician E.I. Chazov of the Ministry of Health of the Russian Federation, Moscow, Russia; ^3^Laboratory of Gas Exchange, Biomechanics and Barophysiology, State Scientific Center of the Russian Federation—The Institute of Biomedical Problems of the Russian Academy of Sciences, Moscow, Russia; ^4^Department of Innovative Pharmacy, Medical Devices and Biotechnology, Moscow Institute of Physics and Technology, Moscow, Russia

**Keywords:** von Willebrand factor, ADAMTS-13, cardiovascular disease, Heyde syndrome, coronary artery disease

## Abstract

Von Willebrand factor (VWF) is a large multimeric glycoprotein involved in hemostasis. It is essential for platelet adhesion to the subendothelium of the damaged endothelial layer at high shear rates. Such shear rates occur in small-diameter arteries, especially at stenotic sites. Moreover, VWF carries coagulation factor VIII and protects it from proteolysis in the bloodstream. Deficiency or dysfunction of VWF predisposes to bleeding. In contrast, an increase in the concentration of high molecular weight multimers (HMWM) of VWF is closely associated with arterial thrombotic events. Severe aortic stenosis (AS) or hypertrophic obstructive cardiomyopathy (HOCM) can deplete HMWM of VWF and lead to cryptogenic, gastrointestinal, subcutaneous, and mucosal bleeding. Considering that VWF facilitates primary hemostasis and a local inflammatory response at high shear rates, its dysfunction may contribute to the development of coronary artery disease (CAD) and its complications. However, current diagnostic methods do not allow for an in-depth analysis of this contribution. The development of novel diagnostic techniques, primarily microfluidic, is underway. Such methods can provide physiologically relevant assessments of VWF function at high shear rates; however, they have not been introduced into clinical practice. The development and use of agents targeting VWF interaction with the vessel wall and/or platelets may be reasonable in prevention of CAD and its complications, given the prominent role of VWF in arterial thrombosis.

## Introduction

Von Willebrand factor (VWF) owes its name to a Finnish physician Erik von Willebrand, who described an inherited bleeding disorder in 1924, which was later called Von Willebrand disease (VWD). In the 1950s it became clear that the disease was due to a deficiency of some blood protein. This protein was first purified in the early 1970s and its complete sequence was described in 1986 (1). Later research showed that VWF function is shear-rate dependent and is crucial for primary hemostasis in arteries (2, 3). Recent years have seen a surge in studies of VWF role in a number of pathologies other than VWD, as well as introduction of anti-VWF agents.

VWF is a large multimeric glycoprotein that is involved in hemostasis (4). It is essential for platelet adhesion to the subendothelium of the damaged endothelial layer at high shear rates occurring in small-diameter arteries, especially at stenotic sites. Moreover, VWF carries coagulation factor VIII and protects it from proteolysis in the bloodstream. Unprotected by VWF, coagulation factor VIII is unstable and degrades rapidly.

## The structure and functions of the von Willebrand factor

**FIGURE 1:**
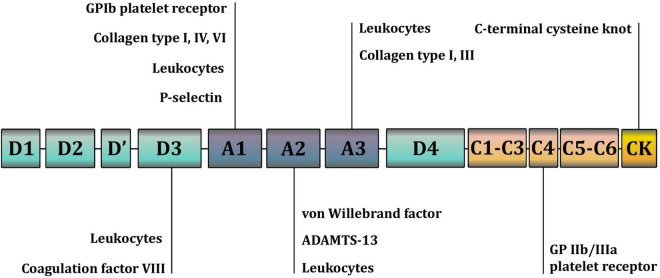
A schematic representation of the domains and binding sites of the Von Willebrand factor (VWF) monomer. The A1 domain contains a binding site for the glycoprotein complex Ib/IX/V (GP Ib) of platelets; collagen types I, IV, VI; and heparin. The A2 domain contains binding sites for the ADAMTS-13 (a disintegrin and metalloproteinase with a thrombospondin type 1 motif 13) metalloprotease and VWF molecules. The A3 domain contains binding sites for collagen types I and III. The C4 domain contains a binding site for the platelet integrin αIIbβ3 (GPIIb/IIIa). C domains facilitate VWF flexibility. The D’D3 domain contains a binding site for coagulation factor VIII. The C-terminal cysteine knot (CTCK) is involved in the dimerization of VWF. All D domains are involved in the formation of disulfide bonds between VWF dimers.

**FIGURE 2:**
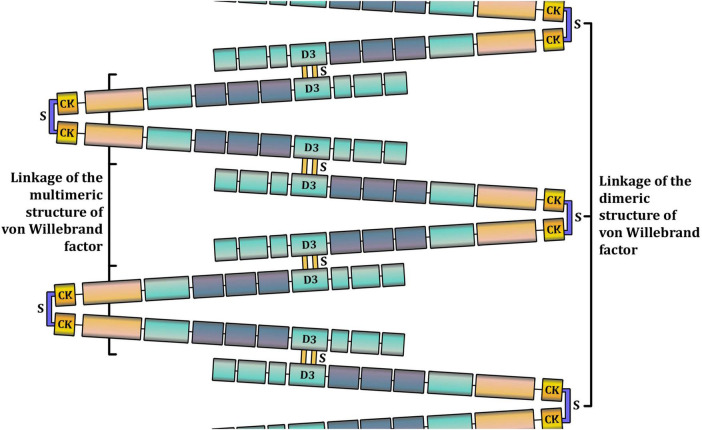
A schematic representation of the multimeric structure of Von Willebrand factor (VWF). In the bloodstream, VWF is present in a range of multimers, from dimers (around 500 kDa) to high-molecular weight multimers (10,000 kDa and more). The larger the VWF multimer is, the more hemostatically active it is. VWF monomers are dimerized in the C-terminal cysteine knot (CTCK) through disulfide bonds. Other disulfide bonds bind VWF dimers in D domains to form multimers.

VWF is a large, complex multimeric protein composed of a varying number of dimers that polymerize into high-molecular-weight multimers (HMWM). The more dimers a VWF molecule contains, the more potent it is in causing hemostasis. Smaller multimers mainly function as carriers of coagulation factor VIII, whereas HMWM are involved in cell adhesion (5, 6). Monomers that form dimers consist of functionally different parts, or domains, of VWF (Figures 1, 2). Each domain contains binding sites for specific cells and molecules. Thus, the A1 domain contains a binding site for the glycoprotein complex Ib/IX/V (GP Ib) of platelets, the only receptor on non-activated platelets with a high affinity for VWF. Additionally, the A1 domain contains binding sites for collagen types I, IV, VI, and heparin. The A2 domain contains binding sites for the ADAMTS-13 (a disintegrin and metalloproteinase with a thrombospondin type 1 motif 13) metalloprotease and VWF molecules. The A3 domain contains binding sites for collagen types I and III. The C4 domain contains a binding site for the platelet integrin αIIbβ3 (GPIIb/IIIa). C domains facilitate VWF flexibility. The D′D3 domain contains a binding site for coagulation factor VIII. The C-terminal cysteine knot (CTCK) is involved in the dimerization of VWF. All D domains are involved in the formation of disulfide bonds between dimers (7).

VWF is mainly produced in the endothelial cells and is densely packed in endothelial Weibel–Palade bodies with P-selectin. Some VWF is produced by megakaryocytes. When mature platelets detach from megakaryocytes, VWF remains inside α-granules (8, 9). VWF is constantly released into the bloodstream from Weibel-Palade bodies of non-activated endothelial cells (basal secretion). Activation of the endothelium upregulates VWF secretion. Further, up to 20% of VWF may be secreted from α-granules upon platelet activation (10, 11). However, the role of platelet VWF in hemostasis has not been fully established, and studies are limited.

**FIGURE 3:**
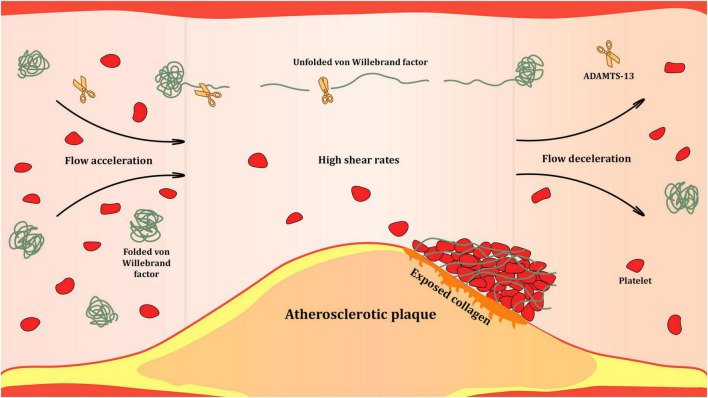
Shear-rate dependent activation of Von Willebrand factor (VWF) at the site of atherosclerotic stenosis. High shear rates occurring in the bloodstream at sites of significant atherosclerotic narrowing of the arterial lumen trigger unfolding of VWF multimers. Unfolded VWF presents a range of binding sites for proteins of subendothelial matrix, platelets and leukocytes. It is also subjected to proteolysis by ADAMTS-13 that cleaves unfolded VWF multimers at the A2 domains and reduces their hemostatic activity.

VWF exists in two forms in the bloodstream: inactive globular and active unfolded (Figure 3), which are determined by the shear rate of the flowing blood. Blood is a heterogeneous liquid, and as it flows through the vessels, it encounters an internal friction force, causing different layers to move at different velocities. The velocity of the flow at the center of the vessel is higher than that at its walls. The magnitude of the difference in the velocities of adjacent layers is quantitatively expressed as the shear rate, measured in reciprocal seconds (s^–1^). At low shear rates, which occur in the veins or large arteries, VWF remains in the globular form. This form conceals VWF domains and prevents interaction with the circulating platelets. At high shear rates, which occur in the small arteries and arterioles, especially at stenotic sites, VWF unfolds and exposes the domains with binding sites (9, 12).

Unfolded VWF strains can self-associate through the A2 domain and form complex mesh structures that significantly increase the number of platelet binding sites. The ability of the unfolded VWF to self-associate at high shear rates was studied *in vitro* by Zheng et al. (13). Schneider et al. reported the formation of mesh structures from VWF strains that unfolded at high shear rates (2).

Upon unfolding, VWF presents binding sites for ADAMTS-13 on A2 domains. This interaction results in the proteolytic cleavage of HMWM of VWF, reducing its hemostatic activity (14). ADAMTS-13 is synthesized exclusively in stellate cells of the liver, and its plasma level is negatively associated with that of VWF (15).

VWF carries coagulation factor VIII in the bloodstream. This factor is involved in the intrinsic coagulation pathway, interacting with factor IXa as a cofactor to form intrinsic tenase, an enzyme complex that converts inactive coagulation factor X into active Xa. Without VWF, coagulation factor VIII is susceptible to rapid degradation. A globular conformation of VWF protects against proteolysis and prolongs the half-life of coagulation factor VIII, ultimately delivering it to the sites of vascular damage (16).

## Diseases associated with von Willebrand factor and ADAMTS-13 deficiency or dysfunction

VWF deficiency or dysfunction predisposes to bleeding. VWD is a congenital disorder caused by quantitative deficiency and/or qualitative changes in the structure of VWF. VWD is one of the most common hemostatic disorders, with the prevalence of severe cases being 1:10,000 in the general population (17). VWD presents with symptoms similar to those observed in hemophilia such as nasal and gingival bleeding, subcutaneous and muscle hematomas, prolonged skin bleeding, and metrorrhagia. Acquired von Willebrand syndrome is a rare disease associated with acquired quantitative and/or qualitative VWF deficiency in lymphoproliferative (chronic lymphocytic leukemia), myeloproliferative (thrombocythemia), cardiovascular (AS and HOCM), and immunological (hypothyroidism) diseases (18).

The increase in the level of HMWM of VWF due to ADAMTS-13 deficiency causes thrombotic thrombocytopenic purpura (TTP), characterized by microvascular thrombosis and platelet pool depletion (19). Quantitative deficiency of ADAMTS-13 in TTP can develop due to a congenital defect or production of autoantibodies that enhance ADAMTS-13 clearance. Functional deficiency can also develop due to autoantibodies that inhibit ADAMTS-13 activity without affecting its plasma level (20). Thrombosis in TTP may manifest as acute kidney injury, neurological and mental disorders, fever, hemolytic anemia, and thrombocytopenia with purpura.

## Diagnostic testing of deficiency and dysfunction of von Willebrand factor

The diagnosis of VWD requires various tests (21). The VWF plasma level, also known as the von Willebrand factor antigen (VWF:Ag), is measured using an enzyme-linked immunosorbent assay (ELISA), which involves a highly specific antigen-antibody reaction. VWF:Ag measures the concentration of plasma VWF molecules, ranging from dimers to HMWM. Normal values of VWF:Ag range from 50 to 150 IU/dL, but levels can vary over a wide range in the same individual on separate occasions (22). Proteolytic cleavage of HMWM of VWF into smaller multimers impairs its hemostatic function without affecting VWF:Ag values. Therefore, VWF:Ag reflects the total amount of this protein in the blood rather than its functional state.

VWF level in the blood depends on blood group (23). This complicates the distinction between healthy individuals with low VWF levels and those with mild VWD (24). Individuals with blood group O have lower VWF:Ag levels than that of those with other blood groups (25, 26). In a study involving 1,117 blood donors, Gill et al. showed that the mean VWF:Ag level in the donors with blood group O was 74.8 IU/dL. The mean VWF:Ag level in donors with blood groups A (105.9 IU/dL) and B (116.9 IU/dL) was higher. The highest VWF:Ag level was seen in donors with blood group AB (123.3 IU/dL) (27). The half-life of VWF may differ depending on the blood group (11).

Ristocetin cofactor activity assay (VWF:RCo) is the gold standard for assessing VWF activity. In this assay, the antibiotic ristocetin causes the A1 domains of VWF to bind platelet GPIb receptors, inducing platelet agglutination. In VWF:RCo, standardized formalin-fixed or lyophilized platelets are added to a sample of platelet-poor plasma containing VWF and ristocetin. This leads to VWF-dependent platelet agglutination, which is measured using an aggregometer. VWF:RCo induces VWF activation by a non-physiological chemical agent. This test is used to diagnose VWD. It is especially sensitive in severe cases, such as VWD type 3, in which VWF is almost or completely absent from the blood. Studies revealed that normal values of VWF:RCo were blood group-dependent. In a study on 167 healthy donors, Moeller et al. showed that VWF:RCo values were 67.7 ± 19, 81.2 ± 27.7, 95.5 ± 24.5, and 102.3 ± 19.1% in blood groups O, A, B, and AB, respectively (28). In a study on 200 healthy children, Akin et al. demonstrated that VWF:RCo values were 89 ± 23 and 103 ± 17% in blood groups O and non-O, respectively (29).

Structural analysis of VWF multimers using agarose gel electrophoresis remains the gold standard for assessing HMWM deficiency (6, 30). This assessment is critical to the identification of VWD type 2 subtypes. It can also be applied to the diagnosis of HMWM deficiency due to acquired von Willebrand syndrome in patients with severe valvular disease. Additionally, VWF collagen binding assay (VWF:CB) can be used to assess HMWM deficiency (24).

The platelet function analyzer-100 (PFA-100), a device that assesses primary hemostasis, has often been used in VWD screening (31). The analysis is performed in a chamber consisting of a narrow tube and membrane with a 150 μm aperture. A whole blood sample is drawn via the tube and through the aperture into the membrane, and coated with collagen and platelet activators, such as adenosine diphosphate (ADP) and epinephrine, or ADP and prostaglandin E1. The shear rate in the aperture can reach 6,000 s^–1^, which is sufficient to unfold and activate VWF. A PFA-100 test measures the time to occlusion of the aperture by thrombus formation (closure time). This complex process involves numerous interactions that lead to platelet adhesion and aggregation. Therefore, PFA-100 does not allow isolation of the contribution of shear rate-dependent VWF activation to thrombus formation (31). Currently, PFA-100 is often employed in research.

## Von Willebrand factor and cardiovascular disease

### Aortic stenosis

Aortic stenosis (AS) is a valvular heart disease eventually resulting in the left ventricular outflow tract (LVOT) obstruction. The most common cause of AS is chronic inflammatory degeneration and calcification of aortic valve leaflets. Severe AS may result in a number of life-threatening conditions, including heart failure, myocardial ischemia, arrhythmias, and cardiac arrest. The treatment of severe AS, especially after the onset of symptoms, usually involves aortic valve replacement (32).

Cryptogenic gastrointestinal bleeding in patients with AS was first described by Heyde in 1958 (33). In 1992, Warkentin et al. suggested that bleeding from gastrointestinal angiodysplasia was caused by the loss of HMWM of VWF due to AS or hypertrophic cardiomyopathy (HCM) (34). In 2002, Warkentin et al. reported two cases of severe AS with gastrointestinal bleeding that resolved after aortic valve replacement (35). Platelet count, activated partial thromboplastin time, coagulation factor VIII, VWF:Ag level, and VWF:RCo values were normal preoperatively. However, a pronounced reduction in HMWM of VWF was present, which recovered after aortic valve replacement and remained normal during the 10-year follow-up period (35). The level of HMWM of VWF can decrease by 50% in severe AS (36). Moreover, low pulse pressure in AS contributes to a decrease in the endothelial basal secretion of VWF (37, 38).

The primary reason for the loss of HMWM of VWF is that AS increases the shear rate at the aortic valve orifice. As the entire volume of circulating blood passes through the stenotic orifice, all HMWM of VWF are subjected to unfolding and proteolysis by ADAMTS-13. This eventually results in a quantitative deficiency of HMWM of VWF (acquired von Willebrand syndrome type 2A), which manifests as bleeding (39). The combination of AS, acquired von Willebrand syndrome type 2A, and bleeding from gastrointestinal angiodysplasia is known as Heyde syndrome. Additionally, patients with severe AS report subcutaneous and mucosal bleeding. The severity of bleeding increases with AS progression (39). The HMWM of VWF recovers within several hours following surgical valve replacement, which normalizes the shear rate in the valve orifice and pulse pressure (38, 40). The loss of HMWM of VWF does not occur in severe coronary or peripheral artery stenosis, because only a small portion of the blood is subjected to high shear rates.

The loss of HMWM of VWF in severe AS and recovery after aortic valve replacement has been demonstrated in a number of studies. Panzer et al. studied 47 patients with severe AS who underwent aortic valve replacement (41). Initially, the level of HMWM of VWF decreased in all patients. It recovered after aortic valve replacement in most patients. The PFA-100 closure time was prolonged preoperatively and normalized thereafter. The authors showed that the loss of HMWM of VWF affected platelet adhesion and ADP-induced platelet aggregation (41). Vincentelli et al. reported subcutaneous and mucosal hemorrhages in 21% of patients with severe AS (42). The VWF:Ag level was within normal range in all patients. The PFA-100 test demonstrated prolonged closure time. The levels of HMWM of VWF and VWF:CB were reduced. The closure time and level of HMWM of VWF normalized 1 day after valve replacement (42). Patients with severe AS who underwent aortic valve replacement presented the same pattern in a study by Frank et al. (36). The PFA-100 closure time was prolonged, and the level of HMWM of VWF was reduced preoperatively. Both parameters normalized following successful valve replacement. The 18-month follow-up showed that the level of HMWM of VWF did not decrease again after aortic valve replacement. This observation was supported by several other studies with a follow-up period of 2 weeks–6 months (36). However, HMWM of VWF may not be replenished if severe aortic regurgitation occurs after aortic valve replacement (42). Despite bleeding, VWF:Ag and VWF:RCo values were within the normal range in patients with Heyde syndrome (36). Acquired von Willebrand and Heyde syndromes were reported in patients with aortic and mitral regurgitation (38, 43–45).

### Hypertrophic obstructive cardiomyopathy

Hypertrophic obstructive cardiomyopathy (HOCM) is a genetic disorder, most frequently transmitted through an autosomal dominant pattern. It results in asymmetric myocardial hypertrophy of the left ventricle that may lead to LVOT. HOCM is a significant cause of sudden cardiac death, arrhythmias and heart failure in young people. In severe cases, septal reduction therapy either with surgical septal myectomy or alcohol ablation can be used to reduce LVOT (46).

LVOT obstruction exposes HMWM of VWF to proteolysis by ADAMTS-13, similarly to AS. Blackshear et al. studied five patients with symptomatic HOCM (47). Spontaneous gastrointestinal, mucosal, or excessive postoperative bleeding was observed in all patients. VWF: Ag and VWF: RCo values were within normal range, whereas electrophoresis showed the loss of HMWM and an excess of low-molecular weight multimers of VWF. Bleeding ceased, and HMWM of VWF recovered following septal myectomy in all patients (47). In another study of 28 patients with HOCM, the VWF:Ag level was normal in all patients (48). The PFA-100 closure time was prolonged in all but one patient. Loss of HMWM of VWF was detected in all patients. A strong positive correlation was found between the peak pressure gradient in the LVOT and percentage loss of HMWM of VWF, relative to all VWF multimers. The peak pressure gradient 15 mmHg in the LVOT at rest was sufficient to reduce the HMWM of VWF (48).

### Left ventricular assist devices

Left ventricular assist devices (LVAD) are pumping systems used in patients with the end-stage heart failure refractory to medical therapy as a bridge-to-transplant or an alternative to heart transplantation option (49). Gastrointestinal bleeding is among the most common complications associated with LVAD use. Pump construction may cause the loss of HMWM of VWF due to increased shear rates in the LVAD-driven circulation. LVAD explantation is associated with the recovery of HMWM of VWF, within a few hours (50).

### Coronary artery disease

Coronary artery disease (CAD) is a chronic condition characterized by atherosclerotic plaque accumulation in the epicardial arteries. Retention of atherogenic lipoproteins in arterial intima and low-grade vascular inflammation are recognized as the main drivers of atherosclerosis development (51). Oxidation of low-density lipoproteins may link lipoprotein retention and proinflammatory macrophage activation in the vessel wall (52). Recently, the role of mitochondrial DNA mutations was suggested in induction of sterile vascular inflammation (53). Atherosclerotic plaques in epicardial arteries can eventually rupture or erode, resulting in acute coronary syndrome (ACS) in a form of myocardial infarction (MI) or unstable angina. Plaques can also gradually progress to significant narrowing of arterial lumen, causing angina pectoris, heart failure, or remain asymptomatic. CAD usually follows a pattern of long stable periods intermitted by unstable episodes due to atherothrombotic events (54).

Impairment of the hemostatic role of VWF may contribute to the development of CAD and its complications. In addition, VWF may contribute to inflammation in atherosclerosis. VWF facilitates leukocyte recruitment and extravasation at high shear rates (55). VWF may modulate the inflammatory response at the sites of atherosclerotic lesions through this function.

CAD is less prevalent in patients with VWD than in healthy individuals. The prevalence of atherosclerotic cardiovascular disease (CAD, MI, brain ischemia, and peripheral artery disease) was assessed in 7,556 patients with VWD and 19,918,970 patients without VWD (56). The prevalence of CAD was 15.0% in patients with VWD vs. 26.0% in patients without VWD. The risk factor-adjusted odds ratio was 0.86 [95% confidence interval (CI), 0.80–0.94] for CAD and 0.69 (95% CI, 0.61–0.79) for MI in patients with VWD (56).

In a study by Xu et al., the VWF:Ag level, which differed between patients with CAD and healthy individuals, was 141.78 ± 20.53 IU/dL in patients with CAD vs. 111.95 ± 17.15 IU/dL in healthy controls (57). Kaikita et al. reported that the VWF:Ag level was 2,151 ± 97 mU/mL in patients hospitalized within 72 h from the onset of MI, 1,445 ± 93 mU/mL in patients with exertional angina and 90% narrowing of a major coronary artery, and 1,425 ± 76 mU/mL in patients with chest pain without stenotic coronary atherosclerosis on diagnostic cardiac catheterization (58). In contrast, the ADAMTS-13 level was the lowest in patients experiencing acute MI (799 ± 29 mU/mL), and higher in patients experiencing exertional angina (996 ± 31 mU/mL) and in those without significant coronary artery stenosis (967 ± 31 mU/mL) (58). In another study, the VWF:Ag level was almost 1.5-fold higher in 1,026 patients with ST-segment elevation myocardial infarction (STEMI) than in 652 control patients (378.2 ng/mL vs. 264.4 ng/mL, respectively), whereas ADAMTS-13 activity was lower in patients with STEMI than in the controls (90 and 97%, respectively) (59). No association was observed between VWF:Ag and ADAMTS-13 levels in the SMILE study, which included 560 men who experienced a MI at least 6 months prior and 646 healthy men. ADAMTS-13 (101 and 100%, respectively) and VWF:Ag (138 and 135%, respectively) levels did not differ between the groups (60).

The time to recovery of the VWF:Ag level after STEMI was studied in 57 male rats in which the anterior descending artery was permanently ligated approximately 2 mm from its origin (61). The rats were divided into four groups. Blood was initially collected from the coronary sinus and inferior vena cava in all groups, 1 h after the onset of MI in the first group, 24 h after the onset of MI in the second group, 7 days after the onset of MI in the third group. The fourth group was the control sham-operated group. Compared with the initial values, the VWF:Ag level in the blood from the coronary sinus increased 1.31-fold 1 h after MI and 0.88-fold 24 h later. The VWF:Ag level in the blood from the inferior vena cava increased 0.37-fold 1 h after MI and 0.18-fold 24 h later. The VWF:Ag level normalized 7 days after MI (61).

The association between the VWF:Ag level and CAD risk was studied in an initially CAD-free population (62). In a prospective study, 1,411 men without CAD were divided into tertiles depending on the VWF:Ag level, and followed up for 16 years. After adjusting for cardiovascular risk factors, the odds ratio was 1.53 (95% CI, 1.10–2.12) for CAD in the upper tertile when compared with the lower tertile (62). Another prospective study followed up approximately 10,000 healthy men for 5 years (63). CAD developed in 296 patients (158 developed MI and 142 developed stable and unstable angina). The baseline VWF:Ag level was higher in patients who developed MI (129.2 ± 53.1 IU/dL) compared with the control patients (115.9 ± 41.8 IU/dL). The relative risk of MI was 3.04-fold (95% CI, 1.59–5.80) higher in participants with a VWF:Ag level in the upper quartile when compared with the lower quartile (63). The prospective Reykjavik study enrolled 1,925 patients without CAD who subsequently developed MI or fatal CAD during the 19.4-year follow-up and 3,616 controls (64). The baseline VWF:Ag level was higher in patients with major adverse cardiovascular events (MACE) than it was in the control group. After adjusting for cardiovascular risk factors, the increase by one standard deviation above baseline VWF:Ag level corresponded to an odds ratio of 1.08 (95% CI, 1.02–1.15) for MI or lethal CAD (64). However, according to the large ARIC study on 14,477 participants initially free from CAD, an elevated VWF:Ag did not provide added value for cardiovascular risk assessment when adjusted to traditional cardiovascular risk factors (65). Several other studies reported that the VWF:Ag level assessment did not improve predicting MACE when adjusted to traditional cardiovascular risk factors (66–68). Therefore, the low predictive value of the VWF:Ag level in cardiovascular risk assessment in CAD-free individuals can be partially explained by confounding cardiovascular risk factors (69).

Contrary to studies on the general CAD-free population, studies on patients with preexisting CAD found a direct relationship between the VWF:Ag level and MACE rate (70–72). The prospective ECAT study followed up 3,043 patients with angina pectoris for 2 years. Those who subsequently developed MI or sudden cardiac death had higher baseline VWF:Ag levels. Patients were divided into quintiles depending on the VWF:Ag level. The relative risk of MACE in the upper quintile was 1.85-fold higher than it was in the lower quintile (70). The ENTIRE-TIMI 23 study enrolled 314 patients with STEMI who had VWF:Ag levels measured before and 48–72 h after fibrinolysis (71). The study showed that the VWF:Ag level in the upper quartile was associated with a higher incidence of repeated MI and death in the subsequent 30 days, than that in the lower quartile (11.2 and 4.1%, respectively) (71). Another study followed up 123 MI survivors under the age of 70 years for 4.9 years (72). The VWF:Ag level was measured 3 months after the onset of MI. A higher VWF:Ag level was independently associated with recurrent MI and death (72).

A VWF:Ag level increase can be caused by factors that contribute to CAD development, such as age (73, 74) and smoking (73, 75). Patients with diabetes mellitus (DM) had higher VWF:Ag levels than those without DM in the ASCET study (74). According to Stehouwer et al., increased VWF:Ag levels in patients with DM type 2 occurred in response to microalbuminuria (76). Several studies have addressed the relationship between arterial hypertension and the VWF:Ag level. In a study by Lip et al., The VWF:Ag level was higher in hypertensive patients than in healthy controls (113 and 98 IU/dL, respectively) (77). In a study by Lee et al., 73 patients with stable CAD and arterial hypertension and 35 healthy controls underwent 24-h ambulatory blood pressure (BP) monitoring (78). The patients were divided into four groups: The first group included patients with high arterial pulse pressure, the second included those with low arterial pulse pressure, the third included dippers, and the fourth included non-dippers. In all groups the VWF:Ag level was higher than the in the control subjects (197 ± 58 and 120 ± 18 IU/dL, respectively). Patients with high arterial pulse pressure and non-dippers had the highest VWF:Ag level (219 ± 58 and 222 ± 55 IU/dL, respectively) (78).

VWF:Ag levels increase in response to stress. Stress signals induce the release of vasopressin, which stimulates the release of VWF from endothelial Weibel-Palade bodies. Desmopressin, a vasopressin analog, is used to sustain the VWF plasma level in VWD treatment (79). A treatment-induced VWF:Ag level increase can occur due to the use of diuretics, digoxin, unfractionated heparin, and oral anticoagulants (57). Unlike unfractionated heparin, enoxaparin reduces VWF release (71, 80).

CAD can affect the VWF:Ag level. Endothelial dysfunction is crucial to the pathogenesis of atherosclerosis, increasing the risk of MACE (81). Considering the predominantly endothelial source of VWF in the bloodstream, higher VWF:Ag levels in patients with CAD may be associated with characteristic endothelial dysfunction and endothelial damage. An increase in the concentration of circulating VWF can propagate chronic local inflammation of the arterial wall. An increase in VWF:Ag levels in response to inflammation was found in patients with systemic inflammatory diseases. The VWF:Ag level was higher in patients with rheumatoid arthritis, scleroderma, and systemic vasculitis than in healthy controls (82). The VWF:Ag level and VWF:RCo values were higher in patients with systemic lupus erythematosus (primarily in those with serositis) than those in healthy controls. However, higher VWF:Ag levels were not associated with clinical manifestations of the disease, including thrombotic complications (83). The VWF:Ag level increases and decreases simultaneously with C-reactive protein in acute inflammation (84). Various inflammatory agents influence the endothelial secretion of VWF. Interleukin-6 (IL-6), IL-8, and tumor necrosis factor-α significantly stimulate the release of VWF from endothelial Weibel-Palade bodies. IL-6 prevents proteolytic cleavage of VWF by ADAMTS-13 (85).

Most studies that addressed the role of VWF in the development of CAD and its complications used ELISA-based VWF:Ag measurements. This assay cannot provide information on the functional state of VWF. Some studies used VWF:RCo, which activated VWF multimers using a non-physiological chemical agent. These laboratory methods failed to reproduce physiologically relevant conditions for platelet adhesion and aggregation. In recent years, microfluidic systems have been popularized. These devices can model hemodynamic conditions, such as different shear rates, which are characteristic of the arterial or venous bed; turbulent flow; complex vessel anatomy, such as bi- or trifurcations; and vessel narrowing by a stenotic plaque, etc. Among the advantages of these devices is that they require small volumes of blood to obtain reproducible results. Moreover, they can examine individual links of hemostasis such as platelet adhesion in isolation from aggregation (86). In future, these devices may be used to diagnose various hemostatic disorders and to test the effectiveness and dose adjustment of antiplatelet drugs. However, such microfluidic systems are not currently standardized and are uncommon in clinical practice (13, 86–90).

## Advances and prospects for agents targeting von Willebrand factor and ADAMTS-13 in cardiovascular disease

The development and use of agents targeting VWF interaction with the vessel wall and/or platelets may be reasonable in prevention of CAD and its complications, given the prominent role of VWF in arterial thrombosis. Most agents used in the primary and secondary prevention of CAD do not affect VWF or ADAMTS-13. Only heparins interfere with GPIb-mediated platelet adhesion by binding to the A1 domain of VWF (91).

At the turn of the twenty-first century, AJvW-2 and AJW200 antibodies targeting the GPIb binding sites on the A1 domain of VWF were developed. A study on dogs with induced occlusive thrombosis in the left coronary artery compared the efficacy of AJW200 and the GPIIb/IIIa antagonist abciximab in thrombosis prevention. AJW200 inhibited thrombus formation without affecting bleeding time and showed a better safety profile than abciximab (92). Similar results were obtained with AJvW-2 in another canine study (93).

Eto et al. studied the effect of AJvW-2 on platelet aggregation at high shear rates in patients with unstable angina or MI and control subjects (94). Platelet aggregation was 2- and 1.3-fold higher in patients with MI and unstable angina, respectively, than in the controls. AJvW-2 completely inhibited platelet adhesion in all groups (94).

Aptamers targeting GPIb interaction with the A1 domain of VWF have been developed. These are small single-stranded RNA or DNA molecules that are capable of high-affinity binding to a target molecule. The first-generation aptamer, ARC1779, produced dose-dependent inhibition of VWF activity in healthy individuals (95). The antithrombotic effect of ARC1779 was studied in 36 patients who underwent carotid endarterectomy. ARC1779 reduced the availability of A1 domains of VWF and the rate of embolic signals detected using doppler ultrasonography; however, perioperative bleeding and anemia were increased (96). Further research on ARC1779 was halted owing to a lack of funding. A second-generation aptamer, TAGX-0004, inhibited the binding of GPIb to the A1 domain of VWF by 10-fold compared to ARC1779. TAGX-0004 inhibited thrombus formation in a flow chamber by 20-fold compared to ARC1779 (97). The third-generation aptamer, BT200, reduced the availability of the A1 domains of VWF in a dose-dependent manner and prolonged PFA-100 closure time by more than 300 s (98). Additionally, BT200 reduced the availability of the A1 domains of VWF in a dose-dependent manner in 320 patients with ACS (99). Another aptamer, DTRI-031, produced a dose-dependent decrease in platelet adhesion (up to complete inhibition) at high shear rates in a microfluidic system. DTRI-031 prevented arterial thrombosis and facilitated thrombus recanalization in murine and canine models of carotid artery injury (100).

Anfibatide is a reversible platelet GP Ib receptor antagonist. In a murine model of focal cerebral ischemia, anfibatide administration resulted in smaller infarct size, a less severe neurological deficit, and histopathological brain tissue changes compared to sham-treated mice (101). Results of anfibatide administration were comparable to that of the GPIIb/IIIa inhibitor tirofiban; however, anfibatide caused less intracerebral hemorrhage and had shorter bleeding time (101). Similar results have been reported by Chu et al. (102). Anfibatide suppressed ristocetin-induced platelet aggregation without affecting bleeding time or coagulation in 94 healthy individuals (103). Another study comprised 90 patients with non-ST elevation MI who were divided into groups of 30 depending on the anfibatide dose and 30 controls (104). Anfibatide was administered in addition to standard dual antiplatelet therapy. Ristocetin-induced platelet aggregation decreased by 47, 16, and 21%, in the high-, medium-, and low-dose groups, respectively, and by 0% in the placebo group. During the 30-day follow-up, death, MI, and major bleeding were rare and the outcomes were comparable between the anfibatide and placebo groups (104).

ALX-0081 (caplacizumab) is the only agent approved for clinical use that targets the VWF-platelet interaction (105). It is a humanized bivalent nanoparticle that targets the GPIb binding sites on the A1 domains of VWF. It was approved for clinical use in the European Union and United States for the treatment of adult patients with TTP, following the results of the HERCULES trial. In this trial, caplacizumab administration resulted in a lower incidence of the composite endpoint of TTP-related death, recurrent TTP, and thromboembolism (105).

Few studies have investigated the efficacy and safety of caplacizumab in patients with CAD. The efficacy of caplacizumab was studied in 9 patients with CAD who were scheduled for elective percutaneous coronary intervention (PCI) and 11 healthy controls (106). Caplacizumab completely inhibited platelet adhesion to collagen at high shear rates in patients with CAD and in healthy controls. However, complete inhibition of adhesion in the patients with CAD required high doses of caplacizumab. The effectiveness of caplacizumab was unaffected by antithrombotic drugs, including acetylsalicylic acid, clopidogrel, and heparin (106). In a study of 46 patients with stable CAD scheduled for elective PCI, caplacizumab was safe and resulted in complete inhibition of platelet aggregation (107). In 2009, caplacizumab was studied in 380 high-risk patients with ACS scheduled for elective PCI. Compared to the GPIIb/IIIa inhibitor abciximab, caplacizumab was not beneficial in reducing the risk of bleeding and had comparable antithrombotic effectiveness (108).

Administration of recombinant human ADAMTS-13 reduced endothelial dysfunction and improved cardiac remodeling in a murine model of left ventricular pressure overload (109). Another study showed that recombinant human ADAMTS-13 administration reduced infarct size, neutrophil infiltration of the ischemic myocardium, and troponin-I release in a murine model of ischemia/reperfusion injury (110).

A novel agent, Revacept, is a fusion protein of the extracellular domain of GPVI, a major platelet collagen receptor, and the human Fc fragment (111). It coats exposed collagen at the sites of vessel injury and prevents platelet adhesion and subsequent aggregation. Similarly, Revacept may interfere with the binding of VWF to collagen (111). In a murine cerebral ischemia/reperfusion injury model, Revacept administered immediately before reperfusion reduced cerebral infarct size, edema, and inflammation, and did not increase the incidence of intracranial bleeding. However, there were no differences in the recovery of neurological functions 24 h after the onset of stroke between mice treated with Revacept and those treated with fibrinolytic rtPA (111). The ISAR-PLASTER study investigated the safety and efficacy of Revacept in 334 patients with stable CAD scheduled for elective PCI (112). Patients received Revacept at a dose of 160 mg, 80 mg, or placebo in addition to standard antiplatelet therapy. Revacept did not show a benefit over standard therapy regarding the primary endpoint of death or myocardial injury and did not affect the incidence of bleeding at 30 days (112). Currently, a clinical trial on the safety and efficacy of Revacept in patients with symptomatic carotid atherosclerosis is underway (113).

Microlyse, a fusion protein consisting of an antibody fragment targeting the CTCK domain of VWF and the protease domain of urokinase plasminogen activator, was described in 2022 (114). This novel agent triggers destruction of platelet-VWF complexes by plasmin on endothelial cells. Antithrombotic effect of Microlyse was more potent than that of caplacizumab in the murine model of TTP. Microlyse also attenuated thrombocytopenia and tissue damage without affecting hemostasis in a tail-clip bleeding murine model (114).

## Conclusion

Severe AS or HOCM predisposes to a deficiency in HMWM of VWF and leads to gastrointestinal, subcutaneous, or mucosal bleeding. Considering that VWF facilitates primary hemostasis and a local inflammatory response at high shear rates, dysfunction of this protein may putatively contribute to the development of CAD and its complications. However, few methods allow for in-depth analysis of this contribution. The development and use of agents targeting VWF interaction with the vessel wall and/or platelets may be reasonable in prevention of CAD and its complications, given the prominent role of VWF in arterial thrombosis.

## Author contributions

SK and EM: conceptualization. SK and ZG: methodology. EM, SO, IM, and YA: data search and analysis and writing—original draft preparation. SK: data curation and funding acquisition. IM, SK, and ZG: writing—review and editing. SO and YA: visualization. ZG: resources, supervision, and project administration. All authors have read and agreed to the published version of the manuscript.

## Abbreviations

ACS: acute coronary syndrome
ADAMTS-13: a disintegrin and metalloproteinase with a thrombospondin type 1 motif 13
AS: aortic stenosis
CAD: coronary artery disease
CI: confidence interval
DM: diabetes mellitus
DNA: deoxyribonucleic acid
ELISA: enzyme-linked immunosorbent assay
GP: glycoprotein
HMWM: high molecular weight multimers
HOCM: hypertrophic obstructive cardiomyopathy
LVAD: left ventricular assist device
LVOT: left ventricular outflow tract
MACE: major adverse cardiovascular events
MI: myocardial infarction
PCI: percutaneous coronary intervention
STEMI: ST-segment elevation myocardial infarction
TTP: thrombotic thrombocytopenic purpura
VWD: von Willebrand disease
VWF: von Willebrand factor
VWF:Ag: von Willebrand factor antigen assay
VWF:CB: von Willebrand factor collagen binding assay
VWF:RCo: ristocetin cofactor activity assay.
